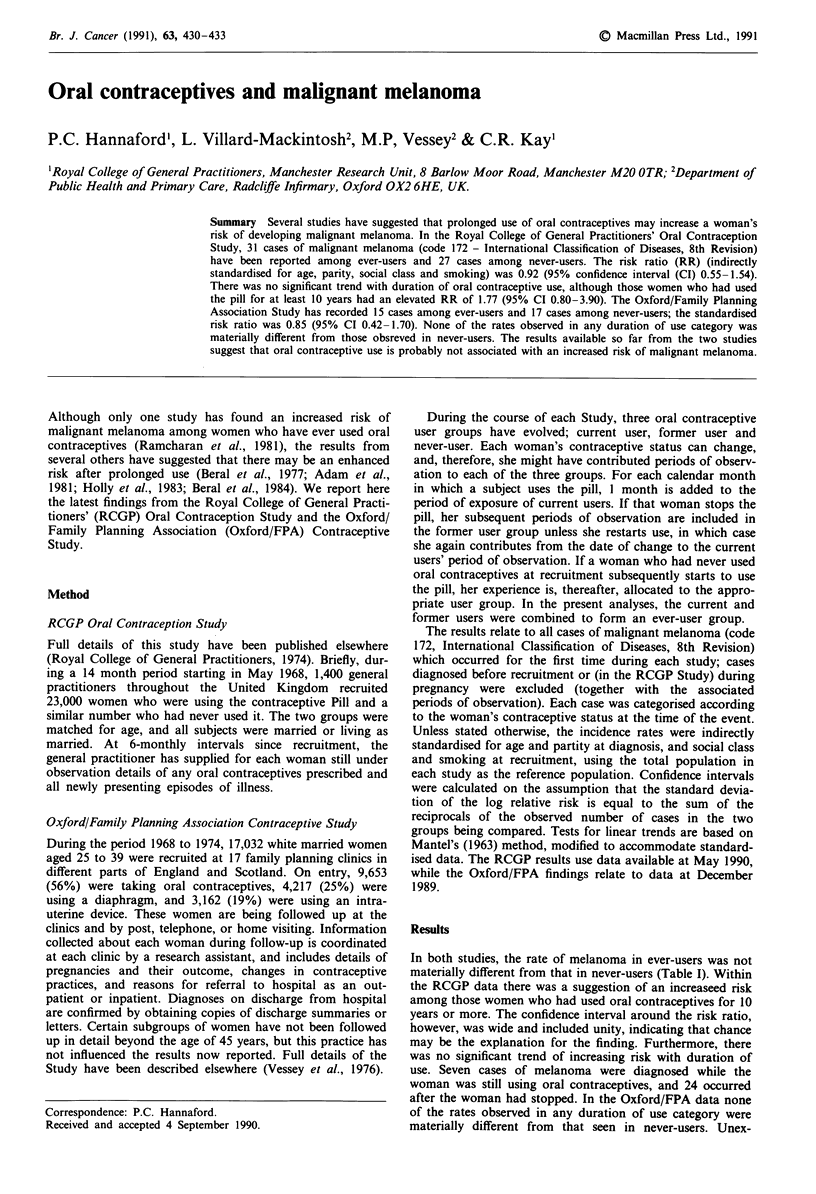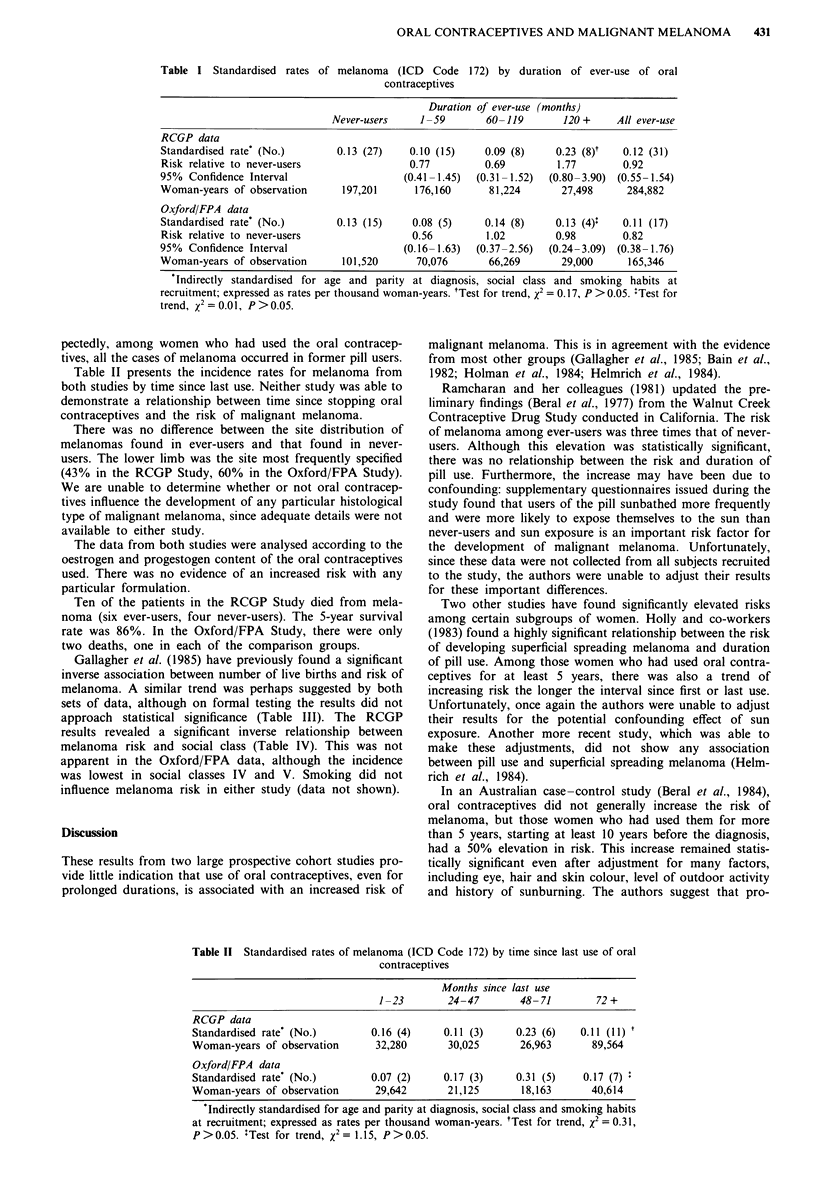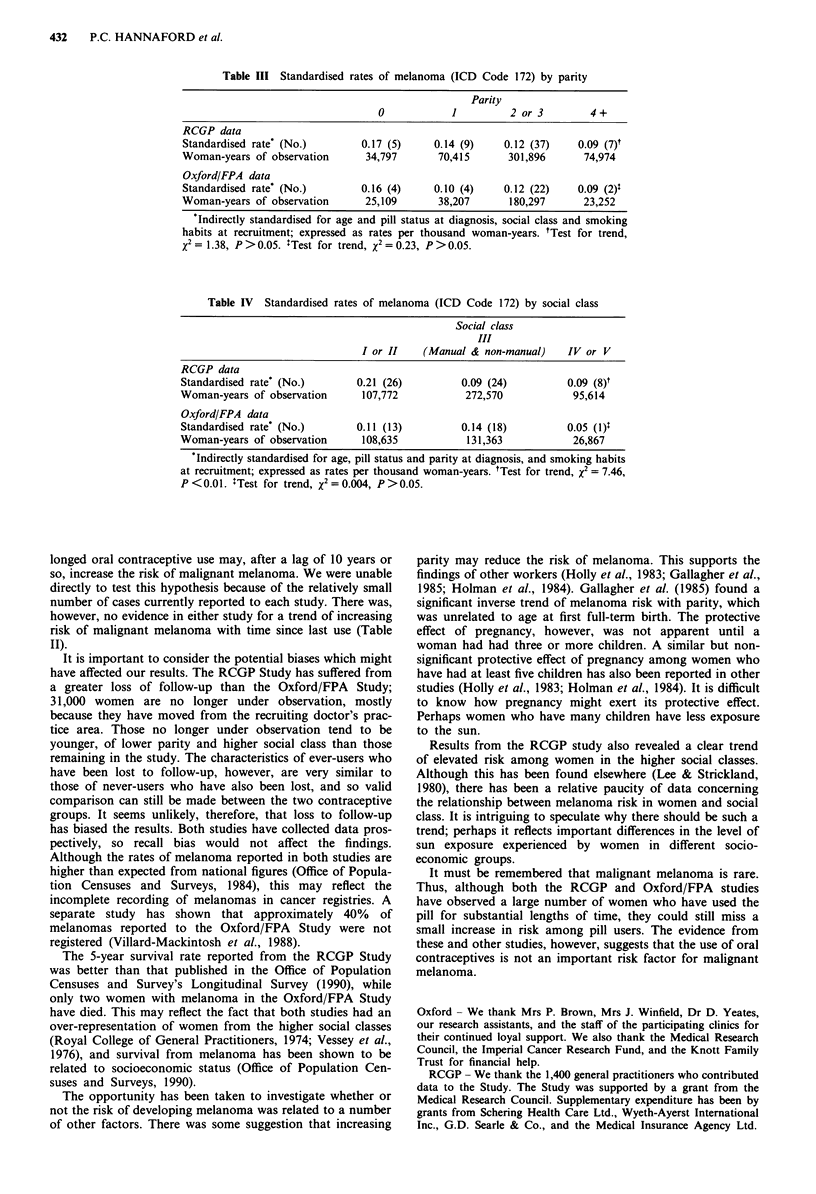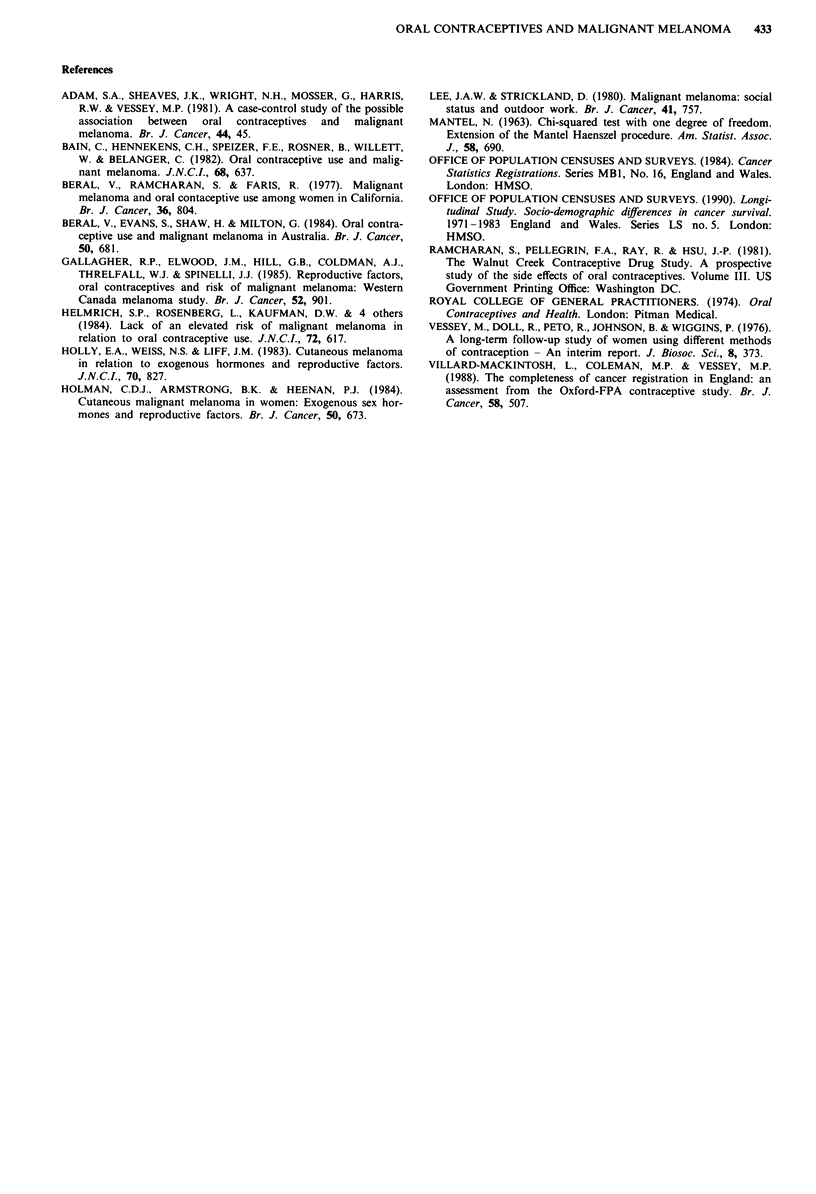# Oral contraceptives and malignant melanoma.

**DOI:** 10.1038/bjc.1991.99

**Published:** 1991-03

**Authors:** P. C. Hannaford, L. Villard-Mackintosh, M. P. Vessey, C. R. Kay

**Affiliations:** Royal College of General Practitioners, Manchester Research Unit, UK.

## Abstract

Several studies have suggested that prolonged use of oral contraceptives may increase a woman's risk of developing malignant melanoma. In the Royal College of General Practitioners' Oral Contraception Study, 31 cases of malignant melanoma (code 172--International Classification of Diseases, 8th Revision) have been reported among ever-users and 27 cases among never-users. The risk ratio (RR) (indirectly standardised for age, parity, social class and smoking) was 0.92 (95% confidence interval (CI) 0.55-1.54). There was no significant trend with duration of oral contraceptive use, although those women who had used the pill for at least 10 years had an elevated RR of 1.77 (95% CI 0.80-3.90). The Oxford/Family Planning Association Study has recorded 15 cases among ever-users and 17 cases among never-users; the standardised risk ratio was 0.85 (95% CI 0.42-1.70). None of the rates observed in any duration of use category was materially different from those observed in never-users. The results available so far from the two studies suggest that oral contraceptive use is probably not associated with an increased risk of malignant melanoma.


Br. J. Cancer (1991), 63, 430–433 © Macmillan Press Ltd., 1991

### Oral contraceptives and malignant melanoma

P.C. Hannaford[1], L. Villard-Mackintosh[2], M.P, Vessey[2] & C.R. Kay[1]

[1]*Royal College of General Practitioners, Manchester Research Unit, 8 Barlow Moor Road, Manchester M20 0TR;* [2]*Department of Public Health and Primary Care, Radcliffe Infirmary, Oxford OX2 6HE, UK.*

**Summary** Several studies have suggested that prolonged use of oral contraceptives may increase a woman's risk of developing malignant melanoma. In the Royal College of General Practitioners' Oral Contraception Study, 31 cases of malignant melanoma (code 172 – International Classification of Diseases, 8th Revision) have been reported among ever-users and 27 cases among never-users. The risk ratio (RR) (indirectly standardised for age, parity, social class and smoking) was 0.92 (95% confidence interval (CI) 0.55–1.54). There was no significant trend with duration of oral contraceptive use, although those women who had used the pill for at least 10 years had an elevated RR of 1.77 (95% CI 0.80–3.90). The Oxford/Family Planning Association Study has recorded 15 cases among ever-users and 17 cases among never-users; the standardised risk ratio was 0.85 (95% CI 0.42–1.70). None of the rates observed in any duration of use category was materially different from those obsreved in never-users. The results available so far from the two studies suggest that oral contraceptive use is probably not associated with an increased risk of malignant melanoma.

Although only one study has found an increased risk of malignant melanoma among women who have ever used oral contraceptives (Ramcharan *et al.*, 1981), the results from several others have suggested that there may be an enhanced risk after prolonged use (Beral *et al.*, 1977; Adam *et al.*, 1981; Holly *et al.*, 1983; Beral *et al.*, 1984). We report here the latest findings from the Royal College of General Practitioners' (RCGP) Oral Contraception Study and the Oxford/Family Planning Association (Oxford/FPA) Contraceptive Study.

### Method

### *RCGP Oral Contraception Study*

Full details of this study have been published elsewhere (Royal College of General Practitioners, 1974). Briefly, during a 14 month period starting in May 1968, 1,400 general practitioners throughout the United Kingdom recruited 23,000 women who were using the contraceptive Pill and a similar number who had never used it. The two groups were matched for age, and all subjects were married or living as married. At 6-monthly intervals since recruitment, the general practitioner has supplied for each woman still under observation details of any oral contraceptives prescribed and all newly presenting episodes of illness.

### *Oxford/Family Planning Association Contraceptive Study*

During the period 1968 to 1974, 17,032 white married women aged 25 to 39 were recruited at 17 family planning clinics in different parts of England and Scotland. On entry, 9,653 (56%) were taking oral contraceptives, 4,217 (25%) were using a diaphragm, and 3,162 (19%) were using an intrauterine device. These women are being followed up at the clinics and by post, telephone, or home visiting. Information collected about each woman during follow-up is coordinated at each clinic by a research assistant, and includes details of pregnancies and their outcome, changes in contraceptive practices, and reasons for referral to hospital as an outpatient or inpatient. Diagnoses on discharge from hospital are confirmed by obtaining copies of discharge summaries or letters. Certain subgroups of women have not been followed up in detail beyond the age of 45 years, but this practice has not influenced the results now reported. Full details of the Study have been described elsewhere (Vessey *et al.*, 1976).

Correspondence: P.C. Hannaford.
Received and accepted 4 September 1990.

During the course of each Study, three oral contraceptive user groups have evolved; current user, former user and never-user. Each woman's contraceptive status can change, and, therefore, she might have contributed periods of observation to each of the three groups. For each calendar month in which a subject uses the pill, 1 month is added to the period of exposure of current users. If that woman stops the pill, her subsequent periods of observation are included in the former user group unless she restarts use, in which case she again contributes from the date of change to the current users' period of observation. If a woman who had never used oral contraceptives at recruitment subsequently starts to use the pill, her experience is, thereafter, allocated to the appropriate user group. In the present analyses, the current and former users were combined to form an ever-user group.

The results relate to all cases of malignant melanoma (code 172, International Classification of Diseases, 8th Revision) which occurred for the first time during each study; cases diagnosed before recruitment or (in the RCGP Study) during pregnancy were excluded (together with the associated periods of observation). Each case was categorised according to the woman's contraceptive status at the time of the event. Unless stated otherwise, the incidence rates were indirectly standardised for age and partity at diagnosis, and social class and smoking at recruitment, using the total population in each study as the reference population. Confidence intervals were calculated on the assumption that the standard deviation of the log relative risk is equal to the sum of the reciprocals of the observed number of cases in the two groups being compared. Tests for linear trends are based on Mantel's (1963) method, modified to accommodate standardised data. The RCGP results use data available at May 1990, while the Oxford/FPA findings relate to data at December 1989.

### Results

In both studies, the rate of melanoma in ever-users was not materially different from that in never-users (Table I). Within the RCGP data there was a suggestion of an increaseed risk among those women who had used oral contraceptives for 10 years or more. The confidence interval around the risk ratio, however, was wide and included unity, indicating that chance may be the explanation for the finding. Furthermore, there was no significant trend of increasing risk with duration of use. Seven cases of melanoma were diagnosed while the woman was still using oral contraceptives, and 24 occurred after the woman had stopped. In the Oxford/FPA data none of the rates observed in any duration of use category were materially different from that seen in never-users. Unex-

**Table I** Standardised rates of melanoma (ICD Code 172) by duration of ever-use of oral contraceptives

| | *Never-users* | *Duration of ever-use (months)* 1–59 | 60–119 | 120+ | *All ever-use* |
|---|---|---|---|---|---|
| *RCGP data* | | | | | |
| Standardised rate* (No.) | 0.13 (27) | 0.10 (15) | 0.09 (8) | 0.23 (8)† | 0.12 (31) |
| Risk relative to never-users | | 0.77 | 0.69 | 1.77 | 0.92 |
| 95% Confidence Interval | | (0.41–1.45) | (0.31–1.52) | (0.80–3.90) | (0.55–1.54) |
| Woman-years of observation | 197,201 | 176,160 | 81,224 | 27,498 | 284,882 |
| *Oxford/FPA data* | | | | | |
| Standardised rate* (No.) | 0.13 (15) | 0.08 (5) | 0.14 (8) | 0.13 (4)‡ | 0.11 (17) |
| Risk relative to never-users | | 0.56 | 1.02 | 0.98 | 0.82 |
| 95% Confidence Interval | | (0.16–1.63) | (0.37–2.56) | (0.24–3.09) | (0.38–1.76) |
| Woman-years of observation | 101,520 | 70,076 | 66,269 | 29,000 | 165,346 |

*Indirectly standardised for age and parity at diagnosis, social class and smoking habits at recruitment; expressed as rates per thousand woman-years. †Test for trend, $\chi^2 = 0.17$, $P > 0.05$. ‡Test for trend, $\chi^2 = 0.01$, $P > 0.05$.

pectedly, among women who had used the oral contraceptives, all the cases of melanoma occurred in former pill users.

Table II presents the incidence rates for melanoma from both studies by time since last use. Neither study was able to demonstrate a relationship between time since stopping oral contraceptives and the risk of malignant melanoma.

There was no difference between the site distribution of melanomas found in ever-users and that found in never-users. The lower limb was the site most frequently specified (43% in the RCGP Study, 60% in the Oxford/FPA Study). We are unable to determine whether or not oral contraceptives influence the development of any particular histological type of malignant melanoma, since adequate details were not available to either study.

The data from both studies were analysed according to the oestrogen and progestogen content of the oral contraceptives used. There was no evidence of an increased risk with any particular formulation.

Ten of the patients in the RCGP Study died from melanoma (six ever-users, four never-users). The 5-year survival rate was 86%. In the Oxford/FPA Study, there were only two deaths, one in each of the comparison groups.

Gallagher *et al.* (1985) have previously found a significant inverse association between number of live births and risk of melanoma. A similar trend was perhaps suggested by both sets of data, although on formal testing the results did not approach statistical significance (Table III). The RCGP results revealed a significant inverse relationship between melanoma risk and social class (Table IV). This was not apparent in the Oxford/FPA data, although the incidence was lowest in social classes IV and V. Smoking did not influence melanoma risk in either study (data not shown).

### Discussion

These results from two large prospective cohort studies provide little indication that use of oral contraceptives, even for prolonged durations, is associated with an increased risk of malignant melanoma. This is in agreement with the evidence from most other groups (Gallagher *et al.*, 1985; Bain *et al.*, 1982; Holman *et al.*, 1984; Helmrich *et al.*, 1984).

Ramcharan and her colleagues (1981) updated the preliminary findings (Beral *et al.*, 1977) from the Walnut Creek Contraceptive Drug Study conducted in California. The risk of melanoma among ever-users was three times that of never-users. Although this elevation was statistically significant, there was no relationship between the risk and duration of pill use. Furthermore, the increase may have been due to confounding: supplementary questionnaires issued during the study found that users of the pill sunbathed more frequently and were more likely to expose themselves to the sun than never-users and sun exposure is an important risk factor for the development of malignant melanoma. Unfortunately, since these data were not collected from all subjects recruited to the study, the authors were unable to adjust their results for these important differences.

Two other studies have found significantly elevated risks among certain subgroups of women. Holly and co-workers (1983) found a highly significant relationship between the risk of developing superficial spreading melanoma and duration of pill use. Among those women who had used oral contraceptives for at least 5 years, there was also a trend of increasing risk the longer the interval since first or last use. Unfortunately, once again the authors were unable to adjust their results for the potential confounding effect of sun exposure. Another more recent study, which was able to make these adjustments, did not show any association between pill use and superficial spreading melanoma (Helmrich *et al.*, 1984).

In an Australian case–control study (Beral *et al.*, 1984), oral contraceptives did not generally increase the risk of melanoma, but those women who had used them for more than 5 years, starting at least 10 years before the diagnosis, had a 50% elevation in risk. This increase remained statistically significant even after adjustment for many factors, including eye, hair and skin colour, level of outdoor activity and history of sunburning. The authors suggest that pro-

**Table II** Standardised rates of melanoma (ICD Code 172) by time since last use of oral contraceptives

| | *Months since last use* 1–23 | 24–47 | 48–71 | 72+ |
|---|---|---|---|---|
| *RCGP data* | | | | |
| Standardised rate* (No.) | 0.16 (4) | 0.11 (3) | 0.23 (6) | 0.11 (11) † |
| Woman-years of observation | 32,280 | 30,025 | 26,963 | 89,564 |
| *Oxford/FPA data* | | | | |
| Standardised rate* (No.) | 0.07 (2) | 0.17 (3) | 0.31 (5) | 0.17 (7) ‡ |
| Woman-years of observation | 29,642 | 21,125 | 18,163 | 40,614 |

*Indirectly standardised for age and parity at diagnosis, social class and smoking habits at recruitment; expressed as rates per thousand woman-years. †Test for trend, $\chi^2 = 0.31$, $P > 0.05$. ‡Test for trend, $\chi^2 = 1.15$, $P > 0.05$.

**Table III** Standardised rates of melanoma (ICD Code 172) by parity

| | Parity | | | |
|---|---|---|---|---|
| | *0* | *1* | *2 or 3* | *4+* |
| *RCGP data* | | | | |
| Standardised rate* (No.) | 0.17 (5) | 0.14 (9) | 0.12 (37) | 0.09 (7)† |
| Woman-years of observation | 34,797 | 70,415 | 301,896 | 74,974 |
| *Oxford/FPA data* | | | | |
| Standardised rate* (No.) | 0.16 (4) | 0.10 (4) | 0.12 (22) | 0.09 (2)‡ |
| Woman-years of observation | 25,109 | 38,207 | 180,297 | 23,252 |

*Indirectly standardised for age and pill status at diagnosis, social class and smoking habits at recruitment; expressed as rates per thousand woman-years. †Test for trend, $\chi^2 = 1.38$, $P > 0.05$. ‡Test for trend, $\chi^2 = 0.23$, $P > 0.05$.

**Table IV** Standardised rates of melanoma (ICD Code 172) by social class

| | Social class | | |
|---|---|---|---|
| | *I or II* | *III (Manual & non-manual)* | *IV or V* |
| *RCGP data* | | | |
| Standardised rate* (No.) | 0.21 (26) | 0.09 (24) | 0.09 (8)† |
| Woman-years of observation | 107,772 | 272,570 | 95,614 |
| *Oxford/FPA data* | | | |
| Standardised rate* (No.) | 0.11 (13) | 0.14 (18) | 0.05 (1)‡ |
| Woman-years of observation | 108,635 | 131,363 | 26,867 |

*Indirectly standardised for age, pill status and parity at diagnosis, and smoking habits at recruitment; expressed as rates per thousand woman-years. †Test for trend, $\chi^2 = 7.46$, $P < 0.01$. ‡Test for trend, $\chi^2 = 0.004$, $P > 0.05$.

longed oral contraceptive use may, after a lag of 10 years or so, increase the risk of malignant melanoma. We were unable directly to test this hypothesis because of the relatively small number of cases currently reported to each study. There was, however, no evidence in either study for a trend of increasing risk of malignant melanoma with time since last use (Table II).

It is important to consider the potential biases which might have affected our results. The RCGP Study has suffered from a greater loss of follow-up than the Oxford/FPA Study; 31,000 women are no longer under observation, mostly because they have moved from the recruiting doctor's practice area. Those no longer under observation tend to be younger, of lower parity and higher social class than those remaining in the study. The characteristics of ever-users who have been lost to follow-up, however, are very similar to those of never-users who have also been lost, and so valid comparison can still be made between the two contraceptive groups. It seems unlikely, therefore, that loss to follow-up has biased the results. Both studies have collected data prospectively, so recall bias would not affect the findings. Although the rates of melanoma reported in both studies are higher than expected from national figures (Office of Population Censuses and Surveys, 1984), this may reflect the incomplete recording of melanomas in cancer registries. A separate study has shown that approximately 40% of melanomas reported to the Oxford/FPA Study were not registered (Villard-Mackintosh *et al.*, 1988).

The 5-year survival rate reported from the RCGP Study was better than that published in the Office of Population Censuses and Survey's Longitudinal Survey (1990), while only two women with melanoma in the Oxford/FPA Study have died. This may reflect the fact that both studies had an over-representation of women from the higher social classes (Royal College of General Practitioners, 1974; Vessey *et al.*, 1976), and survival from melanoma has been shown to be related to socioeconomic status (Office of Population Censuses and Surveys, 1990).

The opportunity has been taken to investigate whether or not the risk of developing melanoma was related to a number of other factors. There was some suggestion that increasing parity may reduce the risk of melanoma. This supports the findings of other workers (Holly *et al.*, 1983; Gallagher *et al.*, 1985; Holman *et al.*, 1984). Gallagher *et al.* (1985) found a significant inverse trend of melanoma risk with parity, which was unrelated to age at first full-term birth. The protective effect of pregnancy, however, was not apparent until a woman had had three or more children. A similar but non-significant protective effect of pregnancy among women who have had at least five children has also been reported in other studies (Holly *et al.*, 1983; Holman *et al.*, 1984). It is difficult to know how pregnancy might exert its protective effect. Perhaps women who have many children have less exposure to the sun.

Results from the RCGP study also revealed a clear trend of elevated risk among women in the higher social classes. Although this has been found elsewhere (Lee & Strickland, 1980), there has been a relative paucity of data concerning the relationship between melanoma risk in women and social class. It is intriguing to speculate why there should be such a trend; perhaps it reflects important differences in the level of sun exposure experienced by women in different socio-economic groups.

It must be remembered that malignant melanoma is rare. Thus, although both the RCGP and Oxford/FPA studies have observed a large number of women who have used the pill for substantial lengths of time, they could still miss a small increase in risk among pill users. The evidence from these and other studies, however, suggests that the use of oral contraceptives is not an important risk factor for malignant melanoma.

Oxford – We thank Mrs P. Brown, Mrs J. Winfield, Dr D. Yeates, our research assistants, and the staff of the participating clinics for their continued loyal support. We also thank the Medical Research Council, the Imperial Cancer Research Fund, and the Knott Family Trust for financial help.

RCGP – We thank the 1,400 general practitioners who contributed data to the Study. The Study was supported by a grant from the Medical Research Council. Supplementary expenditure has been by grants from Schering Health Care Ltd., Wyeth-Ayerst International Inc., G.D. Searle & Co., and the Medical Insurance Agency Ltd.

### References

ADAM, S.A., SHEAVES, J.K., WRIGHT, N.H., MOSSER, G., HARRIS, R.W. & VESSEY, M.P. (1981). A case-control study of the possible association between oral contraceptives and malignant melanoma. *Br. J. Cancer*, **44**, 45.

BAIN, C., HENNEKENS, C.H., SPEIZER, F.E., ROSNER, B., WILLETT, W. & BELANGER, C. (1982). Oral contraceptive use and malignant melanoma. *J.N.C.I.*, **68**, 637.

BERAL, V., RAMCHARAN, S. & FARIS, R. (1977). Malignant melanoma and oral contaceptive use among women in California. *Br. J. Cancer*, **36**, 804.

BERAL, V., EVANS, S., SHAW, H. & MILTON, G. (1984). Oral contraceptive use and malignant melanoma in Australia. *Br. J. Cancer*, **50**, 681.

GALLAGHER, R.P., ELWOOD, J.M., HILL, G.B., COLDMAN, A.J., THRELFALL, W.J. & SPINELLI, J.J. (1985). Reproductive factors, oral contraceptives and risk of malignant melanoma: Western Canada melanoma study. *Br. J. Cancer*, **52**, 901.

HELMRICH, S.P., ROSENBERG, L., KAUFMAN, D.W. & 4 others (1984). Lack of an elevated risk of malignant melanoma in relation to oral contraceptive use. *J.N.C.I.*, **72**, 617.

HOLLY, E.A., WEISS, N.S. & LIFF, J.M. (1983). Cutaneous melanoma in relation to exogenous hormones and reproductive factors. *J.N.C.I.*, **70**, 827.

HOLMAN, C.D.J., ARMSTRONG, B.K. & HEENAN, P.J. (1984). Cutaneous malignant melanoma in women: Exogenous sex hormones and reproductive factors. *Br. J. Cancer*, **50**, 673.

LEE, J.A.W. & STRICKLAND, D. (1980). Malignant melanoma: social status and outdoor work. *Br. J. Cancer*, **41**, 757.

MANTEL, N. (1963). Chi-squared test with one degree of freedom. Extension of the Mantel Haenszel procedure. *Am. Statist. Assoc. J.*, **58**, 690.

OFFICE OF POPULATION CENSUSES AND SURVEYS. (1984). *Cancer Statistics Registrations*. Series MB1, No. 16, England and Wales. London: HMSO.

OFFICE OF POPULATION CENSUSES AND SURVEYS. (1990). *Longitudinal Study. Socio-demographic differences in cancer survival.* 1971–1983 England and Wales. Series LS no. 5. London: HMSO.

RAMCHARAN, S., PELLEGRIN, F.A., RAY, R. & HSU, J.-P. (1981). The Walnut Creek Contraceptive Drug Study. A prospective study of the side effects of oral contraceptives. Volume III. US Government Printing Office: Washington DC.

ROYAL COLLEGE OF GENERAL PRACTITIONERS. (1974). *Oral Contraceptives and Health*. London: Pitman Medical.

VESSEY, M., DOLL, R., PETO, R., JOHNSON, B. & WIGGINS, P. (1976). A long-term follow-up study of women using different methods of contraception – An interim report. *J. Biosoc. Sci.*, **8**, 373.

VILLARD-MACKINTOSH, L., COLEMAN, M.P. & VESSEY, M.P. (1988). The completeness of cancer registration in England: an assessment from the Oxford-FPA contraceptive study. *Br. J. Cancer*, **58**, 507.